# Predictors of the failure of conservative treatment in patients with a thoracolumbar burst fracture

**DOI:** 10.1186/s13018-020-02044-3

**Published:** 2020-11-10

**Authors:** Ehsan Alimohammadi, Seyed Reza Bagheri, Paniz Ahadi, Sahar Cheshmehkaboodi, Homa Hadidi, Shokofeh Maleki, Alireza Abdi

**Affiliations:** 1 Department of Neurosurgery, Imam Reza Hospital, Kermanshah University of Medical Sciences, Kermanshah, Iran; 2 Kermanshah University of Medical Sciences, Imam Reza Hospital, Kermanshah, Iran; 3 Clinical Research Development Center, Taleghani and Imam Ali Hospitals, Kermanshah University of Medical Sciences, Kermanshah, Iran; 4 Nursing and Midwifery School, Imam Reza Hospital, Kermanshah University of Medical Sciences, Kermanshah, Iran

**Keywords:** Burst fracture, Thoracolumbar, Conservative treatment, Risk factors

## Abstract

**Background:**

There is a controversy about the management of patients with a thoracolumbar burst fracture. Despite the success of the conservative treatment in most of the cases, some patients failed the conservative treatment. The present study aimed to evaluate risk factors for the need for surgery during the follow-up period in these patients.

**Methods:**

We retrospectively evaluated 67 patients with a traumatic thoracolumbar burst fracture who managed conservatively at our center between May 2014 and May 2019. Suggested variables as potential risk factors for the failure of conservative treatment including age, gender, body mass index (BMI), smoking, diabetes, vertebral body compression rate (VBCR), percentage of anterior height compression (PAHC), Cobb angle, interpedicular distance (IPD), canal compromise, and pain intensity as visual analog scale (VAS) were compared between patients with successful conservative treatment and those with failure of non-operative management.

**Results:**

There were 41 males (61.2%) and 26 females (38.8%) with the mean follow-up time of 15.52 ± 5.30 months. Overall, 51 patients (76.1%) successfully completed conservative treatment. However, 16 cases (23.9%) failed the non-operative management. According to the binary logistic regression analysis, only age (risk ratio [RR], 2.21; 95% confidence interval [95%], 1.78–2.64; *P* = 0.019) and IPD (RR 1.97; 95% CI 1.61–2.33; *P* = 0.005) were the independent risk factors for the failure of the non-operative management.

**Conclusions:**

Our results showed that older patients and those with greater interpedicular distance are at a higher risk for failure of the conservative treatment. As a result, a closer follow-up should be considered for them.

## Background

Traumatic vertebral body fractures of the thoracolumbar spine are common spinal injuries [1, 2]. Burst fractures compromise about 20% of all the thoracolumbar fractures [3]. These fractures are defined as those that involve both anterior and middle columns of the spine [4, 5]. There is a controversy on the management of thoracolumbar burst fractures [4, 6, 7]. Both surgical and conservative managements have own pros and cons [1, 8]. A shorter period of bed rest, early correction of the deformity, and avoidance of later kyphotic deformity are some advantages of surgical treatment [9, 10]. On the other hand, avoidance of surgical procedures with its risks and morbidity as well as decreasing associated costs should be considered as advantages of conservative management [7, 11]. However, some studies demonstrated that there is no significant difference between conservative or surgical management of patients with stable thoracolumbar burst fractures based on pain, functional outcomes, and return to work [6].

Although conservative treatment is successful in most of the cases, some studies reported the failure of conservative treatment in about 20% of these patients [12].

The present study aimed to evaluate the probable risk factors for the failure of conservative treatment in patients with a stable thoracolumbar burst fracture.

## Methods

In the present study, we evaluated 67 consecutive patients with a single level acute traumatic thoracolumbar burst fracture who managed conservatively at our center between May 2014 and May 2019. The inclusion criteria of the study were as follows: (1) a single-level traumatic thoracolumbar burst fracture, (2) age more than 18 years and less than 65 years on admission, (3) the Thoracolumbar Injury Classification and Severity Score (TLICS) less than four on admission.

Patients with any neurological deficits and those with a history of a lumbar surgery as well as those with pathologic or osteoporotic fractures were excluded. Moreover, we excluded patients with multiple fractures.

This study was approved by the ethics committee of our institute. Written informed consent was obtained from all patients. All patients underwent a complete physical examination on admission to the emergency department. Severity scores were calculated for each patient according to the TLICS algorithm [13]. A stable burst fracture was defined as those with a TLICS score < 4. Patients with TLICS score more than 4 on admission were operated and were not included in the study.

Complete radiological evaluations including anteroposterior and lateral thoracolumbar graphy as well as thoracolumbar CT scan were performed for all patients. Moreover, T1- and T2-weighted images and short-tau inversion-recovery (STIR) sequences were used to evaluate the integrity of the posterior ligamentous complex (PLC) (supraspinous ligament, interspinous ligament, ligamentum flavum, facet joint capsule) in each patient [14].

The conservative treatment compromised immobilization, pain relief, and thromboembolism prophylaxis.

A thoracolumbosacral orthosis (TLSO) was prepared by an expert for each subject. Complete bed rest was advised for 2 days. Patients were mobilized after applying the TLSO at the end of the second post traumatic day. Patients were trained to wear TLSO at all times except during resting on the bed for 12 weeks. The VAS was used to assess back pain.

**Fig. 1:**
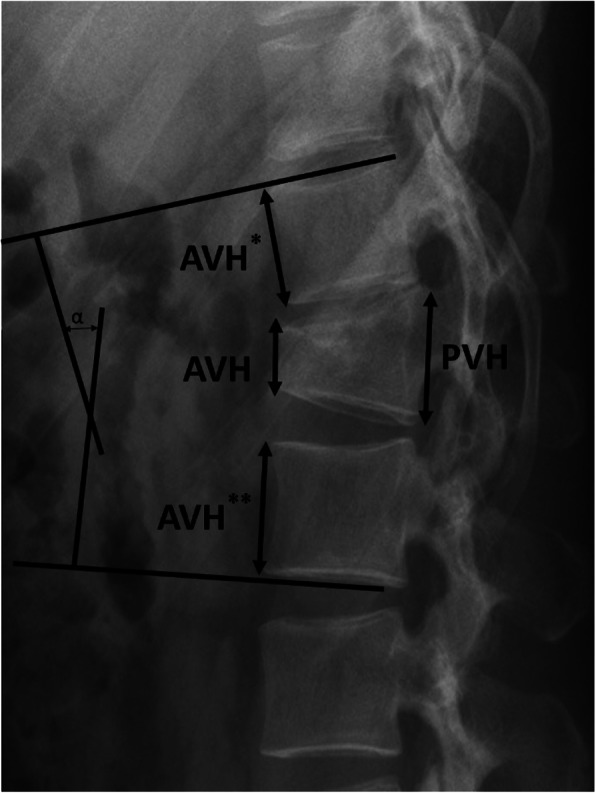
Cobb angle was measured as the angle between the superior endplate of the vertebra above the fracture and the inferior endplate of the vertebra below the fracture. Vertebral body compression rate (VBCR) and percentage of anterior height compression were calculated as follows: VBCR = AVH/PVH × 100%. PAHC = AVH/[(AVH* + AVH**)/2] × 100%. AVH: Anterior vertebral height of the fractured vertebra, AVH*: Anterior vertebral height of a vertebra above the fracture, AVH**: Anterior vertebral height of a vertebra below the fracture, PVH: posterior vertebral height of fractured vertebra

The radiological parameters including vertebral body compression rate (VBCR), percentage of anterior height compression (PAHC), Cobb angle, interpedicular distance (IPD), and canal compromise were calculated as follows:

Cobb angle was measured as the angle between the superior endplate of the vertebra above the fracture and the inferior endplate of the vertebra below the fracture [15] (Fig. 1).

Canal compromise is measured as a ratio of the canal area at the fracture level to the average of the spinal canal diameter at the adjacent vertebrae above and below the fracture [12].

**Fig. 2:**
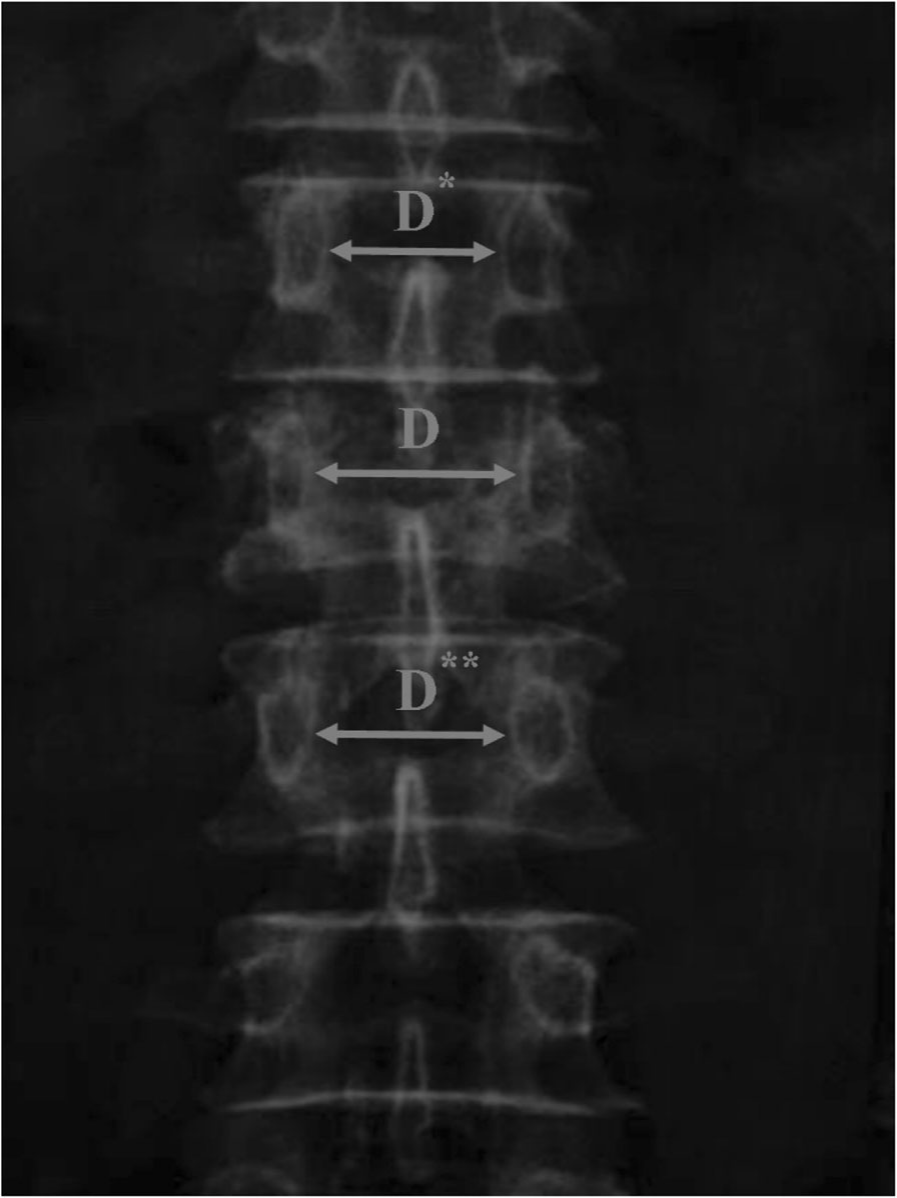
The interpedicular distance (IPD) was calculated by comparing the widening between the pedicles of the fractured vertebrae with the mean of similar values obtained from levels above and below them; IPD = [2D − (D* + D**)/(D* + D**)] × 100%

The interpedicular distance (IPD) was calculated by comparing the widening between the pedicles of the fractured vertebrae with the mean of similar values obtained from levels above and below them [16] (Fig. 2).

Vertebral body compression rate (VBCR) and percentage of anterior height compression(PAHC) were calculated as that shown in Fig. 1 [17].

The failure of conservative treatment was defined as the need for surgery due to progressive neurological deficits and progressive kyphosis during the follow-up period [12].

### Statistical analysis

The data analysis was performed using the SPSS 21 software (SPSS Inc. Chicago, IL).

Data are presented as mean ± standard deviation. We used the Student’s *t* test and the chi-square test for comparisons of continuous and categorical variables between the two groups, respectively. Finally, a binary logistic regression analysis was conducted to determine the independent risk factors for the failure of conservative treatment. A *p* values < 0.05 were considered as the significant level.

## Results

We evaluated 67 patients with a single-level traumatic thoracolumbar burst fracture. There were 41 males (61.2%) and 26 females (38.8%). The mean of age and follow up time were 38.95 ± 11.79 years and 15.52 ± 5.30 months, respectively. The most common cause of trauma was traffic road accidents (55.2%), followed by fall from height (31.3%). Of a total of 67 thoracolumbar fractures, 25 fractures (37.3%) were located at T12 and 19 ones (28.4.2%) were located at L1. Moreover, L2, T11, and T10 were the affected vertebra in 11(16.4%), 8 (11.9%), and 4 (6.0%) cases, respectively (Tables 1 and 2).

**Table 1:** Descriptive characteristics of the sample

Variable	Frequency (%)
Sex	
Male	41 (61.2)
Female	26 (38.8)
Need for surgery	
Yes	16 (23.9)
No	51(76.1)
Cause of injury	
Road traffic	37 (55.2)
Fall	21 (31.3)
Sport	3 (4.5)
Assault	4 (6.0)
Other	2 (3.0)
Level of vertebra	
T10	4 (6.0)
T11	8 (11.9)
T12	25(37.3)
L1	19 (28.4)
L2	11 (16.4)
Smoking	
Yes	26 (38.8)
No	41 (61.2)
Diabetes	
Yes	14 (20.9)
No	53 (79.1)

**Table 2:** Mean and standard deviation of quantitative variables

Variable	Mean	Standard deviation	Range
Age	38.95	11.79	22–65
Follow up	15.52	5.30	7–31
Body mass index	24.73	2.24	21.1–30.9
VBCR (%)	67.31	5.37	62–81
PAHC (%)	71.70	5.01	64–83
Cobb(°)	13.10	4.13	8–24
Canal compromise (%)	23.28	4.71	15–33
IPD(%)	19.82	6.49	13–31
VAS	5.71	0.79	4–8

*VBCR* vertebral body compression rate, *PAHC* percentage of anterior height compression, *IPD* interpedicular distance, *VAS* visual analog scale

As shown in Tables 1 and 2, 26 cases (38.8%) were smokers and 14 subjects (20.9%) suffered from diabetes mellitus. The mean and standard deviations of BMI and VAS were 24.73 ± 2.24 Kg and 5.71 ± 0.79.

Overall, 51 patients (76.1%) completed conservative treatment. However, 16 cases (23.9%) failed non-operative treatment and needed a surgical procedure during the follow-up period. Progressive neurological deficits and progressive kyphosis during the follow-up period were the cause of the need for surgery in 3 (18.75%) and 13 (81.25%) cases, respectively. The posterior transpedicular instrumentation and fusion was performed for 15 subjects (93.75%). One patient (6.25%) needed a combined anterior-posterior approach.

### Risk factors for the need for surgery by univariate analysis

Older patients, those with a higher BMI, smokers, cases with greater cobb angle, and those with a higher interpedicular distance in fractured vertebra had a higher risk for the need for surgery according to the univariate analysis(*p* < 0.05) (Tables 3 and 4).

**Table 3:** Relationship between qualitative variables and needs to surgery

Variable	Need for surgery		Statistical analysis
	Yes *N* (%)	No *N* (%)	
Sex			
Male	10 (24.3)	31 (75.7)	*p* = 0.947
Female	6 (23.0)	20 (77.0)	
Cause of injury			
Road traffic	10 (27.1)	27 (72.9)	N/A
Fall	6 (28.2)	15 (71.8)	
Sport	0 (0.0)	3(100)	
Assault	0 (0.0)	4 (100)	
Other	0(0.0)	2 (100)	
Level of Vertebra			
T10	1 (25.0)	3 (75.0)	N/A
T11	3 (37.5)	5 (62.5)	
T12	7 (28.0)	18 (72.0)	
L1	3 (15.7)	16 (84.3)	
L2	2 (18.1)	9 (81.9)	
Smoking			
Yes	12 (46.1)	14 (53.9)	*p **= 0.001**
No	4 (9.7)	37 (90.3)	
Diabetes			
Yes	5 (31.2)	11 (69.8)	*p* = 0.306
No	9 (17.6)	42 (82.4)

**Table 4:** Relationship between need for surgery and quantitative variables

Variable	Need for surgery		Statistical test
	Yes	No	
Age (years)	53.37 (11.09)	34.43 (7.7)	*p **< 0.001**
Follow up	18.62 (4.68)	14.54 (5.01)	*p* = 0.327
Body mass index	27.37 (2.47)	23.9 (1.3)	*p **= 0.009**
VBCR (%)	63.51 (1.36)	68.52 (4.49)	*p* = 0.401
PAHC (%)	66.68 (1.70)	71.27 (4.21)	*p* = 0.390
Cobb(°)	18.06 (3.64)	11.54 (3.13)	*p **= 0.012**
Canal compromise (%)	26.31 (3.57)	22.33 (4.6)	*p* = 0.187
IPD	29.18 (4.91)	16.68 (2.53)	*p **< 0.001**
VAS	6.31 (0.87)	5.52 (0.67)	*p* = 0.289

*VBCR* vertebral body compression rate, *PAHC* percentage of anterior height compression, *IPD* interpedicular distance, *VAS* visual analog scale

**Table 5:** Binary logistic regression analysis

Variables	Risk ratio	95% CI	*p* value
Age	2.21	1.78–2.64	*p **= 0.019**
Smoking	1.61	1.34–1.88	*p* = 0.745
Body mass index	1.30	0.97–1.63	*p* = 0.813
Cobb (°)	1.57	1.17–1.98	*p* = 0.08
IPD	1.97	1.61–2.33	*p **= 0.005**

There was no relationship between the need for surgery and patients’ gender, VBCR, PAHC, canal compromise, or the VAS [Tables 3 and 4].

### Risk factors for the need for surgery by multivariate analysis

According to the binary logistic regression analysis only age (risk ratio [RR], 2.21; 95% confidence interval [95% CI], 1.78–2.64; *p* = 0.019) and IPD (RR, 1.97; 95% CI 1.61–2.33; *p* = 0.005) were the independent risk factors for the need for surgery (Tables 5).

## Discussion

In the present study, of a total of 67 patients with a conservatively managed stable thoracolumbar burst fracture, 16 patients (23.6%) failed non-operative treatment and had to receive surgery. Moreover, according to the results of the binary logistic regression model, age and IPD were independent predictors of the need for surgery.

There is a controversy on the treatment of stable thoracolumbar burst fractures. Some studies reported satisfactory results from the non-operative treatment of these patients [2, 6, 9]. On the other hand, some studies showed a better outcome for surgically treated ones [10]. Aligizakis et al., in a prospective study, investigated the functional outcomes of 60 patients with a conservatively managed burst fracture. After a 42-month follow-up period, functional outcome was satisfactory in 91.65% of their cases. Moreover, they found that the Cobb angle and anterior vertebral body compression rate reduced. However, that reduction was not statistically significant [2].

In a prospective randomized study with a long-term follow-up, Wood et al. compared the long-term outcomes of surgically treated patients with thoracolumbar burst fractures with the clinical outcome of conservatively treated ones. They found that the average kyphosis was not remarkably different between the two groups. However, the non-operative group had a significantly better outcome based on VAS, (ODI), Roland and Morris disability questionnaire and the Short Form-36 (SF-36) health survey [7].

Butler et al. evaluated the functional outcome of 31 patients with burst L1 fracture that treated surgically or conservatively. Their findings showed that conservatively treated patients had a better functional outcome than surgically treated ones. Furthermore, there was no relationship between vertebral collapse or kyphosis and clinical outcome [9].

On the other hand, the results of a multicenter prospective randomized trial conducted by Siebenga et al. showed better clinical outcomes for surgically treated patients. Moreover, the percentage of patients returning to their work was significantly higher in the surgically treated ones [10].

Despite the success of the conservative treatment in most of the patients with stable thoracolumbar burst fractures, some patients failed the conservative treatment during the follow-up period [12, 18]. The present study aimed to evaluate risk factors for the need for surgery during the follow-up period in these patients. Shen et al in a retrospective study evaluated 129 patients with traumatic thoracolumbar fractures. In their study, 25 cases (19.4%) failed to complete non-operative management. The results of the univariate analysis revealed a significant difference between the patients with a successful completed conservative treatment and those needed a surgical operation during the follow-up period based on age, pain intensity on admission, kyphotic angle, and interpedicular distance. However, according to regression analysis, the intensity of pain and IPD were the only predictors of the need for surgery in initially conservatively treated patients [12]. In accordance with their findings, our results showed the IPD as an independent predictor of the need for surgery during the follow-up period. However, in contrast with their results, the intensity of pain on admission had not any relationship with the failure of conservative treatment in our study. Moreover, age was found as an independent risk factor for the need for surgery in our study, while the result of the binary logistic regression model did not show any significant relationship between age and the failure of conservative treatment in their study. The lower bone quality and weaker bone regeneration ability in older patients could be considered as an explanation for the higher risk for incomplete conservative treatment [7, 12].

The IPD was another independent risk factor for the failure of conservative treatment in our study. Some studies suggested the IPD as an important factor for evaluating the severity of thoracolumbar burst fractures [19, 20]. It has been proposed that with the widening of interpedicular distance after trauma, the probability of retropulsion of the bone fragments into the spinal canal and as a result the chance of neurological deficit may be increased [20]. In a retrospective study, Caffaro et al. investigated the diagnostic significance of IPD in thoracolumbar burst fractures and its relationship with spinal canal compromise and with the presence of neurological deficits. They found a significant relationship between the IPD and severity of canal compromises as well as the presence of neurological deficits. They concluded that IPD could be used as a reliable instrument to evaluate the percentage of the spinal canal narrowing, laminar fractures, and the presence of neurological deficits [19].

In our study, according to the univariate analysis the BMI, smoking, and Cobb’s angle also were associated with the failure of the conservative treatment. However, the final logistic model did not show any relationship between these variables and the need for surgery during the follow-up period. It has been shown that smoking can cause increased vertebral and endplate porosity and decreased trabecular thickness [21, 22]. Moreover, smoking increases cortisol, can kill osteoblasts, causes estrogen imbalance, leads to impeding calcitonin, and decreases oxygen supply, and reduces calcium absorption [21].

These changes could lead to increase bone resorption and decrease bone formation and could be a reason for the failure in the conservative treatment. Moreover, smoking could negatively affect spinal fusion healing [21].

Although it has been demonstrated that high BMI may have some protective effect against fracture risk, the structural and biomechanical disadvantages of obesity may be a reason for the failure of the conservative treatment in obese patients [23].

There are some explanations that could explain the relationship between the Cobb’s angle and the failure of conservative treatment. Besides being an indicator for assessing vertebral body collapse, kyphotic angle also affects sagittal alignment [17]. Papaioannou et al. reported that a reduction of 4 cm in vertebral body height could lead to more than 15° of kyphotic deformity [24]. Hsu et al. demonstrated that kyphotic angle may be increased by 1° when there is a height difference of 7 mm between posterior vertebral height and anterior vertebral height [17].

Moreover, some studies showed that the greater the kyphotic angle, the greater the chance of intervertebral cleft occurring at the body of the fractured vertebra [17].

The present study has several limitations. This was a single-center retrospective study without a long term follow-up. Moreover, the sample size was limited. So, we suggest large prospective multicenter trials to investigate the predictors of the failure of conservative treatment in patients with stable thoracolumbar burst fractures.

## Conclusions

Our results showed that older patients and those with greater interpedicular distance in their fractured vertebra are at a higher risk for failure of conservative treatment. As a result, a closer follow-up should be considered for them.

## Data Availability

All data are available from the corresponding author upon reasonable request.

## Abbreviations

BMI: Body mass index
VBCR: Vertebral body compression rate
PAHC: Percentage of Anterior Height Compression
IPD: Interpedicular distance
VAS: Visual analog scale
TLICS: Thoracolumbar Injury Classification and Severity Score
TLSO: Thoracolumbosacral orthosis
